# Erratum

**Published:** 2005-11

In Figure 2D of Greer et al. [ Environ Health Perspect 110:927–937 (2002)], there should have been seven subjects in the 0.007 mg/kg-day group, but *EHP* erroneously included an extra line (without symbols), indicating a nonexistent eighth subject. This error was reproduced in the commentary of Ginsberg and Rice [ Environ Health Perspect 113:1117–1119 (2005)] and was included in their argument that there was an inhibitory effect overall in that dose group.

*EHP* regrets the error.

## Figures and Tables

**Figure 2D:**
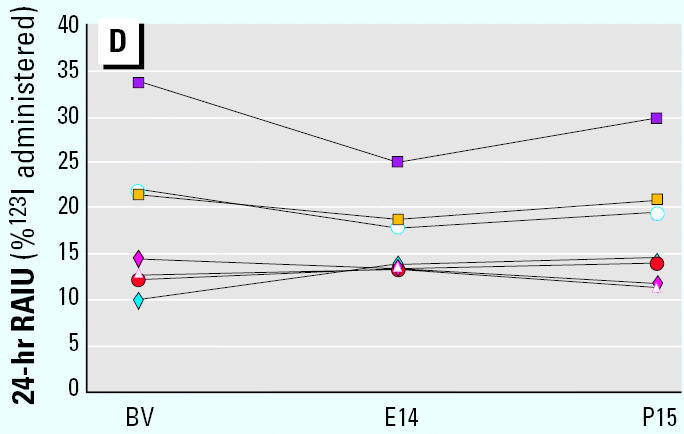
The 24-hr thyroid radioiodine uptake (RAIU) at the baseline visit (BV) and on exposure day 14 (E14) and postexposure day 15 (P15) for each subject in the 0.007-mg/kg-day dose group.

